# Acquired Nisin Resistance in *Staphylococcus aureus* Involves Constitutive Activation of an Intrinsic Peptide Antibiotic Detoxification Module

**DOI:** 10.1128/mSphereDirect.00633-18

**Published:** 2018-12-12

**Authors:** Christopher P. Randall, Arya Gupta, Bret Utley-Drew, Siu Yi Lee, Genevieve Morrison-Williams, Alex J. O’Neill

**Affiliations:** a Antimicrobial Research Centre and School of Molecular and Cellular Biology, Faculty of Biological Sciences, University of Leeds, Leeds, United Kingdom; Antimicrobial Development Specialists, LLC; Uppsala University; University of Bath; Sheffield Hallam University

**Keywords:** bacteriocin, lantibiotic, resistance studies, staphylococci

## Abstract

Resistance to the lantibiotic nisin (NIS) arises readily in Staphylococcus aureus as a consequence of mutations in the *nsaS* gene, which encodes the sensor kinase of the NsaRS two-component regulatory system. Here we present a series of studies to establish how these mutational changes result in reduced NIS susceptibility. Comparative transcriptomic analysis revealed upregulation of the NsaRS regulon in a NIS-resistant mutant of S. aureus versus its otherwise-isogenic progenitor, indicating that NIS resistance mutations prompt gain-of-function in NsaS. Two putative ABC transporters (BraDE and VraDE) encoded within the NsaRS regulon that have been reported to provide a degree of intrinsic protection against NIS were shown to be responsible for acquired NIS resistance; as is the case for intrinsic NIS resistance, NIS detoxification was ultimately mediated by VraDE, with BraDE participating in the signaling cascade underlying VraDE expression. Our study revealed new features of this signal transduction pathway, including that BraDE (but not VraDE) physically interacts with NsaRS. Furthermore, while BraDE has been shown to sense stimuli and signal to NsaS in a process that is contingent upon ATP hydrolysis, we established that this protein complex is also essential for onward transduction of the signal from NsaS through energy-independent means. NIS resistance in S. aureus therefore joins the small number of documented examples in which acquired antimicrobial resistance results from the unmasking of an intrinsic detoxification mechanism through gain-of-function mutation in a regulatory circuit.

**IMPORTANCE** NIS and related bacteriocins are of interest as candidates for the treatment of human infections caused by Gram-positive pathogens such as Staphylococcus aureus. An important liability of NIS in this regard is the ease with which S. aureus acquires resistance. Here we establish that this organism naturally possesses the cellular machinery to detoxify NIS but that the ABC transporter responsible (VraDE) is not ordinarily produced to a degree sufficient to yield substantial resistance. Acquired NIS resistance mutations prompt activation of the regulatory circuit controlling expression of *vraDE*, thereby unmasking an intrinsic resistance determinant. Our results provide new insights into the complex mechanism by which expression of *vraDE* is regulated and suggest that a potential route to overcoming the resistance liability of NIS could involve chemical modification of the molecule to prevent its recognition by the VraDE transporter.

## Abstract (precis)

NIS and related bacteriocins are of interest as candidates for the treatment of human infections caused by Gram-positive pathogens such as Staphylococcus aureus. An important liability of NIS in this regard is the ease with which S. aureus acquires resistance. Here we establish that this organism naturally possesses the cellular machinery to detoxify NIS but that the ABC transporter responsible (VraDE) is not ordinarily produced to a degree sufficient to yield substantial resistance. Acquired NIS resistance mutations prompt activation of the regulatory circuit controlling expression of *vraDE*, thereby unmasking an intrinsic resistance determinant. Our results provide new insights into the complex mechanism by which expression of *vraDE* is regulated and suggest that a potential route to overcoming the resistance liability of NIS could involve chemical modification of the molecule to prevent its recognition by the VraDE transporter.

## INTRODUCTION

Nisin (NIS) is the best-characterized member of a family of bacteriocins known as the lantibiotics and displays potent bactericidal activity against a range of Gram-positive organisms (1). The antibacterial mechanism of action of this agent proceeds via an initial binding event between NIS and the pyrophosphate cage of the peptidoglycan precursor, lipid II, with subsequent insertion of NIS into the cytoplasmic membrane resulting in pore formation and a lethal loss of membrane integrity (2). NIS has been extensively deployed for over 60 years as an antibacterial preservative in food production and is also used in some countries as a topical agent to prevent bovine mastitis (3). Given the dearth of new antibacterial agents receiving approval for systemic use in the treatment of human infections, a number of articles have highlighted the potential for NIS to fill such a role (4–7). Though the compound has a relatively short serum half-life (0.9 h) (8), it has nonetheless been shown to successfully treat staphylococcal and streptococcal infection in mouse models (8, 9).

While NIS may therefore have potential as a chemotherapeutic agent, *in vitro* studies suggest that resistance to this agent can arise readily, a phenomenon that could serve to rapidly compromise its therapeutic utility. In an earlier publication, we demonstrated that substantial reductions (up to 16-fold) in NIS susceptibility could be selected in S. aureus as a consequence of spontaneous mutation (10). At 4× MIC, such NIS-resistant mutants arose at a frequency of ∼2 × 10^−7^ *in vitro*, a figure similar to that seen for antibacterial drugs not generally considered suitable for monotherapy owing to resistance liabilities (10, 11). Further underscoring the idea that these resistant mutants could potentially constitute a threat to therapeutic use of NIS, they proved stable upon extended passage in the absence of selection and resistance was not generally associated with a significant fitness cost *in vitro* (10). The majority of NIS resistant mutants were found to harbor mutations in *nsaS*, a gene encoding the sensor histidine kinase (SHK) portion of a two-component system (TCS) termed NsaRS (also known as BraRS [12]). This TCS has been shown to participate in regulating expression of resistance to the peptidic antibiotic bacitracin and is also one of several such TCS modules in S. aureus that have been reported to provide the bacterium with a degree of intrinsic protection against NIS (10, 12). The mechanism by which mutations in *nsaS* lead to acquired NIS resistance has not been established, and the present study was therefore initiated to gain insight into this phenomenon.

## RESULTS

### NIS resistance mutations lead to constitutive activation of NsaS, resulting in upregulation of the NsaRS regulon.

Following a sensory stimulus, SHKs such as NsaS undergo a conformational change that triggers autophosphorylation of a conserved histidine residue and subsequent phosphotransfer to a conserved aspartate on the response regulator (RR) protein (NsaR in this case) (13). RRs typically act as transcription factors, with activation of gene expression by the RR being dependent upon its phosphorylation state (13). In our previous study, we speculated that NIS resistance mutations in *nsaS* confer a gain of function on the encoded protein (10), with NIS resistance resulting from consequent upregulation of the NsaRS regulon. To exclude the possibility that NIS resistance is instead the result of loss of NsaS function, we disrupted *nsaS* by insertional inactivation in the nisin-resistant strain S. aureus SH1000 NsaS_A208E_ (NIS MIC, 64 mg/liter). Susceptibility testing of the resulting strain revealed complete loss of resistance, with the NIS MIC returning to the same level as that of the NIS-susceptible parent strain (SH1000; 4 mg/liter). This observation implies that NIS resistance is not attributable to a loss of function in NsaS.

To define more precisely the consequences of NIS resistance mutations on NsaS function, RNAseq was employed to compare global gene expression profiles in SH1000 NsaS_A208E_ versus SH1000. Compared with the parent strain, the expression of 16 genes was found to be upregulated ≥2-fold in SH1000 NsaS_A208E_ (Table 1), with 9 genes downregulated ≤2-fold (data not shown). Of the upregulated genes, five (*braD*, *braE*, *vraD*, *vraE*, and *vraH*) are known to be part of the regulon previously shown to be controlled by NsaRS (12, 14), and a further four (SAOUHSC_03040, SAOUHSC_03041, SAOUHSC_03042, and SAOUHSC_03042a/*vraH2*) lie immediately downstream of *vraDEH* on the SH1000 chromosome and likely constitute part of the same operon (14). These results corroborate the idea that NIS resistance mutations in *nsaS* confer a gain in function on the encoded protein, leading to upregulation of its cognate regulon through constitutive activation.

**TABLE 1:** Genes overexpressed ≥2-fold in the NIS-resistant S. aureus strain SH1000 NsaS_A208E_ versus the NIS-susceptible progenitor, SH1000

Locus tag	Encoded protein	Fold change in expression^*a*^
SAOUHSC_00355	Hypothetical protein of unknown function	94
SAOUHSC_01005	Hypothetical protein of unknown function	2.2
SAOUHSC_01068	Hypothetical protein of unknown function	2.4
SAOUHSC_01761	Hypothetical protein of unknown function	2.3
SAOUHSC_01844	Hypothetical protein of unknown function	2.7
SAOUHSC_02745	Hypothetical protein of unknown function	2.6
SAOUHSC_02872	Hypothetical protein of unknown function	5.6
SAOUHSC_02953	Permease domain-containing protein (BraE)	7.0
SAOUHSC_02954	ABC transporter ATP-binding protein (BraD)	7.3
SAOUHSC_03036	ABC transporter ATP-binding protein (VraD)	480
SAOUHSC_03037	Permease domain-containing protein (VraE)	460
SAOUHSC_03037a	Transmembrane protein required for intrinsic daptomycin and gallidermin resistance (VraH)	620
SAOUHSC_03040	Integrase	92
SAOUHSC_03041	Phage tail protein	22
SAOUHSC_03042	Integrase	120
SAOUHSC_03042a	Duplication of SAOUHSC_03037a (VraH2)	75

a Expression values represent the means from three independent biological replicates and are given to two significant figures.

That NIS resistance in S. aureus requires a mutation leading to upregulation of the NsaRS regulon implies that the NIS molecule itself is incapable of sufficient induction of this system to bring about resistance. In support of this idea, we note that a previous study showed only modest induction of NsaRS regulon members at subinhibitory NIS concentrations (12). Furthermore, when we exposed the NIS-susceptible SH1000 strain to a range of subinhibitory NIS concentrations for 60 min, we observed no reduction in NIS susceptibility in a subsequent MIC determination (data not shown). In contrast, the antibiotic bacitracin has been reported to be a potent inducer of the NsaRS regulon (12), and we found that bacitracin preexposure of SH1000 did result in reduced NIS susceptibility; the maximal effect was observed at a bacitracin concentration of 16 mg/liter, which led to an increase in the NIS MIC of SH1000 (32 mg/liter), similar to that observed for SH1000 NsaS_A208E_ (64 mg/liter).

### BraDE and VraDE are essential for, and universally upregulated in, acquired NIS resistance.

**FIG 1:**
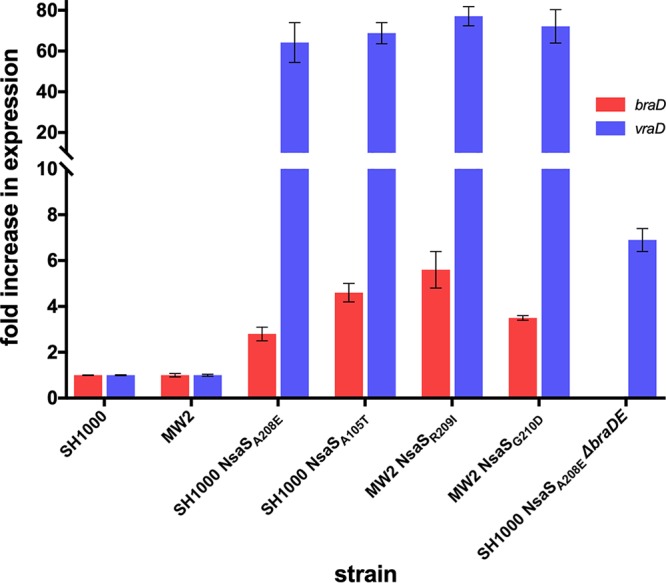
Expression of *braD* and *vraD* in S. aureus strains harboring acquired NIS resistance mutations in *nsaS*. A figure denoting the location of each substitution within the predicted structure of NsaS can be found elsewhere (10). Fold change in expression in NIS-resistant mutants was calculated relative to the corresponding parent strain (SH1000 or MW2) using the ΔΔ*C_T_* method. Values represent the means from at least three independent biological replicates.

We next sought to establish which genes of the NsaRS regulon are responsible for the NIS resistance phenotype of SH1000 NsaS_A208E_. The work of Hiron et al. has established that the *braDE* and *vraDE* genes, which encode two putative ABC transporters, are together capable of providing S. aureus with an intrinsic level of protection against NIS and bacitracin (12). Since both *braDE* and *vraDE* are part of the NsaRS regulon, with both appearing upregulated in the transcriptome analysis of SH1000 NsaS_A208E_ (Table 1), it seemed likely that acquired NIS resistance in the latter strain was attributable to increased *braDE*/*vraDE* expression. We confirmed overexpression of *braDE/vraDE* in SH1000 NsaS_A208E_ using qRT-PCR with oligonucleotide primers specific for *braD* and *vraD*, detecting 2.8 (±0.3)-fold and 64.2 (±9.8)-fold increases in transcription of these genes, respectively, relative to SH1000 (Fig. 1). At the same time, we took advantage of this qRT-PCR approach to establish that NIS resistance mutations encoding substitutions other than NsaS_A208E_ (10) also trigger upregulation of the NsaRS regulon and that the process by which all of these mutations lead to NIS resistance is therefore similar. Comparable levels of *braD*/*vraD* upregulation were observed in S. aureus strains containing NIS resistance mutations encoding NsaS_A105T_, NsaS_R209I_, and NsaS_G210D_ (10) (Fig. 1), confirming that all NsaS polymorphisms associated with NIS resistance result in constitutive activation of this sensor protein and upregulation of the NsaRS regulon.

To establish whether both *braDE* and *vraDE* participate in acquired NIS resistance, we independently deleted *braDE* and *vraDE* in SH1000 NsaS_A208E_ and evaluated the effect on NIS susceptibility. Both Δ*braDE* and Δ*vraDE* mutants of SH1000 NsaS_A208E_ exhibited complete loss of NIS resistance (Table 2), implying that both gene pairs are essential for the acquired NIS resistance phenotype. A series of complementation studies was subsequently undertaken to confirm and further explore this result. NIS resistance was in each case fully restored when the gene pair that had been deleted from the chromosome was provided *in trans* on plasmid pRMC2 (Table 2). In contrast, complementation was unsuccessful when only one gene of the deleted pair was provided *in trans* (Table 2), implying that both components of each putative transporter are required for the acquired NIS resistance phenotype.

**TABLE 2:** NIS susceptibility of SH1000 derivatives^*a*^

Strain	NIS MIC (mg/liter)
SH1000	4
SH1000 (pRMC2:*vraDE*)	64
SH1000 (pRMC2:*braDE*)	4
SH1000 NsaS_A208E_	64
SH1000 NsaS_A208E_ Δ*vraDE*	2
SH1000 NsaS_A208E_ Δ*braDE*	2
SH1000 NsaS_A208E_ Δ*vraDE* (pRMC2:*vraD*)	2
SH1000 NsaS_A208E_ Δ*vraDE* (pRMC2:*vraE*)	2
SH1000 NsaS_A208E_ Δ*vraDE* (pRMC2:*vraDE*)	64
SH1000 NsaS_A208E_ Δ*vraDE* (pRMC2:*braDE*)	2
SH1000 NsaS_A208E_ Δ*braDE* (pRMC2:*braD*)	2
SH1000 NsaS_A208E_ Δ*braDE* (pRMC2:*braE*)	2
SH1000 NsaS_A208E_ Δ*braDE* (pRMC2:*braDE*)	64
SH1000 NsaS_A208E_ Δ*braDE* (pRMC2:*vraDE*)	64
SH1000 NsaS_A208E_ Δ*braDE* (pRMC2:*braD*_E168Q_*E*)	64

a Expression from pRMC2 constructs induced with 0.125 mg/liter anhydrotetracycline.

### Roles of VraDE and BraDE in acquired NIS resistance.

BraDE and VraDE have been shown to play distinct roles in intrinsic resistance to NIS/bacitracin in S. aureus (12). BraDE is thought to participate in sensing these compounds at the membrane and, via an ATP-dependent mechanism, transduce this signal to NsaS (12). Onward transduction of the signal through NsaRS prompts upregulation of VraDE, which is directly responsible for detoxification of these antibiotics through a mechanism that has been postulated to involve transport (either export or import of antibiotic) (12, 14). To confirm that acquired NIS resistance is also ultimately mediated by VraDE alone, we artificially overexpressed VraDE in both SH1000 and SH1000 NsaS_A208E_ Δ*braDE*, in both instances creating strains for which NIS had an MIC of 64 mg/liter (identical to that seen for SH1000 NsaS_A208E_; Table 2). Conversely, artificial overexpression of BraDE in SH1000 and SH1000 NsaS_A208E_ Δ*vraDE* had no impact on NIS susceptibility (Table 2).

The essentiality of BraDE for acquired NIS resistance implies that the existing model for the role of this protein complex in protecting S. aureus from peptide antibiotics is incomplete; under this model, in which BraDE lies upstream of NsaS in the signal transduction pathway, acquired NIS resistance mutations mediating constitutive activation of NsaS would obviate an initial sensing/signaling event by BraDE. Consequently, BraDE must have a function in addition to sensing, leading us to speculate that BraDE assists in some way with the process of onward signal transduction from NsaS. Using qRT-PCR of *vraD* to report on expression of the NsaRS regulon, we sought evidence of such a role for BraDE by comparing *vraD* expression in strains SH1000, SH1000 NsaS_A208E_, and SH1000 NsaS_A208E_ Δ*braDE.* Deletion of *braDE* in SH1000 NsaS_A208E_ caused a substantial (∼9-fold) drop in *vraD* expression (from 64.2- ± 7.3-fold to 6.9- ± 0.5-fold, relative to SH1000), supporting the idea that BraDE is required for optimal signal transduction through NsaRS. In contrast to antibiotic sensing by BraDE, this process is not dependent on ATP hydrolysis by BraD; the NIS resistance phenotype was successfully restored in SH1000 NsaS_A208E_ Δ*braDE* upon expression *in trans* of BraDE carrying an engineered E_168_Q substitution in the Walker B motif of BraD that abolishes ATP hydrolysis by this protein (Table 2).

**FIG 2:**
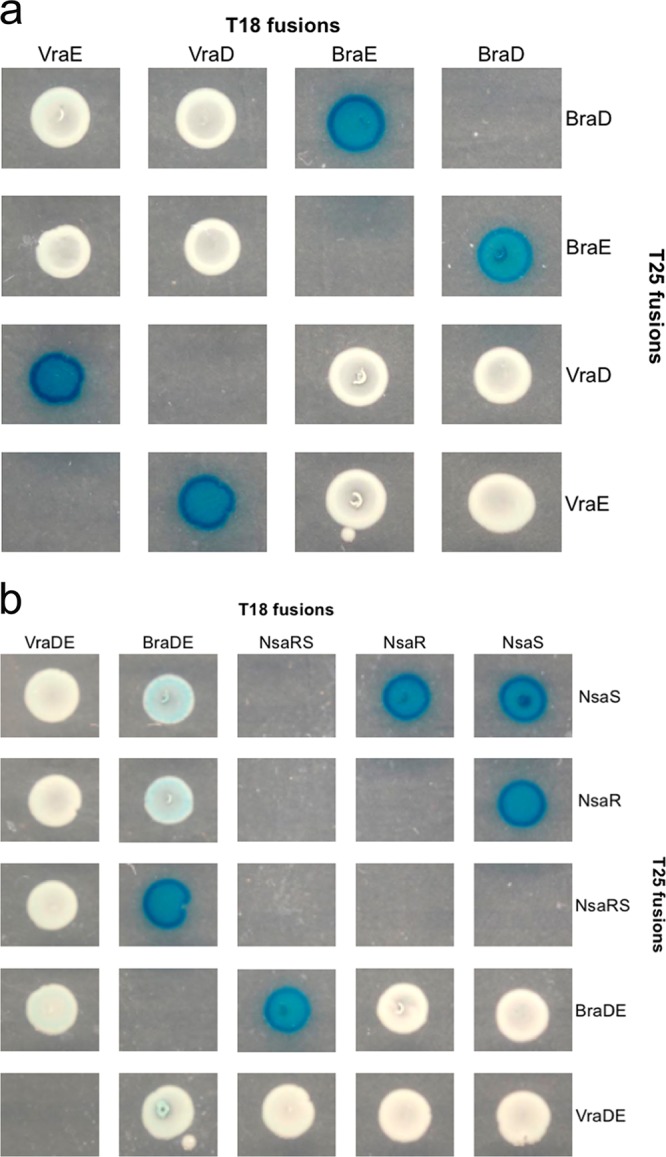
Identification of protein-protein interactions between proteins involved in acquired NIS resistance using bacterial two-hybrid analysis. Protein-protein interactions were tested using the BACTH system, with genes encoding proteins of interest cloned into the pUT18/pUT18C and pKT25/pKNT25 vectors in every conceivable combination. Blue colonies signal a protein-protein interaction, while white colonies imply that no interaction is taking place. Empty boxes represent interactions that were not tested. Results presented are representative of at least three independent experiments.

How does BraDE aid signal transduction through NsaRS? Based on the detailed understanding of TCSs that already exists (13), several steps must occur for successful signal transduction from NsaS to NsaR. These include dimerization of NsaS, recruitment of NsaR to NsaS, and phosphotransfer from NsaS to NsaR. We considered that BraDE might directly associate with NsaS and/or NsaR, thereby acting as a physical scaffold to facilitate one or more of these steps. To explore this possibility, two-hybrid analysis was carried out using the BACTH system to identify physical interactions between these proteins (Fig. 2). Control experiments were first conducted to establish that the system could successfully detect anticipated interactions among proteins of this NIS detoxification module; as expected for the domains of ABC transporters, interaction could be demonstrated between the ATP binding domains, BraD and VraD, and their cognate permeases (BraE and VraE, respectively). BraDE was shown to interact with NsaRS, though a weaker interaction was also detected between BraDE and the individual components of this TCS, NsaS and NsaR (Fig. 2). NsaS was able to interact with itself and NsaR in the BACTH system; since this experiment was conducted in the absence of BraDE, this result implies that BraDE is not required for NsaS dimerization or interaction between NsaS and NsaR.

## DISCUSSION

**FIG 3:**
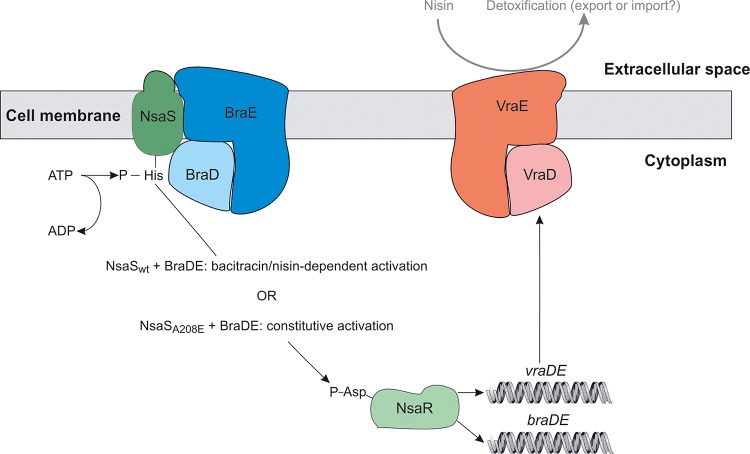
Predicted roles of NsaRS, BraDE, and VraDE in intrinsic and acquired NIS resistance. In the case of intrinsic resistance, the presence of NIS or bacitracin in the extracellular space is detected by the NsaS/BraDE complex, which in turn activates the cognate response regulator NsaR via phosphotransfer to achieve upregulation of BraDE/VraDE expression (12). Detoxification of NIS and bacitracin is ultimately achieved by VraDE through an as-yet-unknown mechanism. In acquired resistance, an amino acid substitution in NsaS (e.g., A_208_E) uncouples NIS sensing from activation of NsaR, leading to constitutively high levels of BraDE/VraDE expression and high-level NIS resistance.

Understanding the mechanisms by which bacterial pathogens resist the action of antibacterial agents constitutes an integral part of the preclinical evaluation of such compounds. Since NIS and related bacteriocins are of considerable interest as candidates for antistaphylococcal chemotherapy in humans (15–17), we sought to dissect the mechanism underlying acquired resistance to NIS in S. aureus. Having previously demonstrated that NIS-resistant strains of S. aureus harbor mutations in the SHK (NsaS) of the NsaSR TCS, we have shown here that these mutations prompt constitutive activation of NsaS and the NsaSR regulon, with resistance resulting ultimately from dramatically upregulated expression of the VraDE transporter (Fig. 3). Thus, while S. aureus naturally possesses the necessary cellular machinery to detoxify NIS, the bacterium is ordinarily sensitive to NIS because this machinery is not expressed at a sufficiently high level to deliver resistance, and the regulatory circuit controlling its expression does not effectively recognize or respond to the presence of NIS (Fig. 3).

Acquired resistance to NIS is therefore one of a small number of examples in which antimicrobial resistance has been shown to arise through the unmasking of an intrinsic detoxification module owing to a gain-of-function mutation in a TCS. Other examples of this phenomenon include enterococcal resistance to teicoplanin (18) and resistance to silver (19) and colistin (20) in *Enterobacteriaceae*, with resistance mutations in each case identified in the corresponding SHK (VanS_B_, SilS/CusS, and PmrB, respectively) that result in constitutive expression of the resistance determinant (18–20). It remains to be established precisely how these mutations—including the NIS resistance mutations found in NsaS—result in the uncoupling of signaling from sensing in the TCS. However, by analogy with previously characterized TCS proteins (e.g., EnvZ, NtrB, PhoQ, and NarX), these mutations typically lie in regions of the SHK essential for phosphatase activity (21–23); loss of phosphatase activity would effectively trap an SHK in the kinase state, leading to constitutive phosphorylation of the RR even in the absence of an inducing stimulus.

NsaS lacks an extracytoplasmic sensing domain and is, in the absence of a gain-of-function mutation, reliant on BraDE for substrate detection (12). Regulatory systems in which an ABC transporter acts as the sensing module for an SHK are not unusual among the *Firmicutes*, with BceAB/BceRS from Bacillus subtilis providing the archetypal example (24). Substrate sensing by such ABC transporters occurs via a large extracytoplasmic domain, with the resulting stimulus transduced to the SHK through a poorly understood mechanism that is contingent upon ATP hydrolysis (12, 25). This signal transduction event is believed to involve a protein-protein interaction between the transporter and the SHK (26, 27); our demonstration that BraDE physically interacts with both NsaS and NsaR corroborates the idea that sensing ABC transporters directly associate with their cognate SHK proteins.

We have also shown that BraDE plays an essential role in NIS resistance in addition to the initial sensing event, participating in onward transduction of the signal from activated NsaS via a mechanism that does not require the hydrolysis of ATP. Given that NsaS appears to be competent for dimerization and subsequent interaction with NsaR in the absence of BraDE (Fig. 2), we propose that BraDE is instead acting to enable phosphotransfer from NsaS to NsaR. In potential support of this idea, accessory regulator proteins that interact with SHKs and stimulate kinase activity at the level of phosphotransfer have previously been described (28). While the distinct roles of BraDE (sensing, downstream signal transduction from the SHK) have therefore been independently observed among the proteins of other regulatory circuits, there is to our knowledge no reported precedent for an ABC transporter that does both. Future work should clarify whether this reflects a common but as-yet-undiscovered feature of other sensing ABC transporters or if BraDE is unique in this regard.

The ease with which NIS resistance is selected and maintained in S. aureus constitutes a potential threat to its efficacy in the treatment of bovine mastitis and represents an important liability to be considered in the context of advancing NIS and related bacteriocins toward therapeutic deployment in humans. Recognition that the VraDE transporter is ultimately responsible for mediating most acquired NIS resistance in S. aureus suggests that a potential route to overcoming this resistance liability could involve modification of the NIS molecule to prevent VraDE recognizing it as a substrate. Toward this end, we note that numerous natural variants and engineered derivatives of NIS have been described in the literature to date, with even limited chemical changes in the NIS molecule achieving considerable modulation of its biological properties (29).

## MATERIALS AND METHODS

### Bacterial strains, culture conditions, and susceptibility testing.

S. aureus and Escherichia coli strains (Table 3) were routinely cultured in Mueller-Hinton broth (MHB) and lysogeny broth (LB), respectively, at 37°C with vigorous aeration. Where appropriate, cultures were supplemented with ampicillin (100 mg/liter), chloramphenicol (10 mg/liter), or erythromycin (5 mg/liter) to maintain plasmids. The MIC of NIS against S. aureus strains was determined by broth microdilution in MHB according to the CLSI method (30). MIC determinations following antibiotic preexposure were performed in an identical manner, with the exception that actively growing cultures were first exposed to a doubling dilution series of subinhibitory concentrations of nisin or bacitracin for 1 h.

**TABLE 3:** Bacteria and plasmids used in this study

Strain or plasmid	Description	Reference(s) or source
Bacterial strains		
S. aureus SH1000	Derivative of strain 8325-4, containing functional *rsbU*	37, 38
S. aureus SH1000 (NsaS_A208E_)	NIS-resistant derivative of SH1000	10
S. aureus SH1000 (NsaS_A105T_)	NIS-resistant derivative of SH1000	10
S. aureus MW2	Community-acquired MRSA strain	3
S. aureus MW2 (NsaS_R209I_)	NIS-resistant derivative of MW2	10
S. aureus MW2 (NsaS_G210D_)	NIS-resistant derivative of MW2	10
E. coli SA08B	Cloning host that modifies cloned DNA for introduction into wild-type S. aureus strains	34
E. coli BTH101	Host strain for two-hybrid assays	36
Plasmids		
pRMC2	E. coli/S. aureus shuttle vector containing the *P*_xyl/tet_ promoter for tetracycline-inducible gene expression in S. aureus	35
pIMAY	E. coli/S. aureus shuttle vector, for allelic replacement in S. aureus	33
pMUTIN4	Suicide vector for insertional inactivation of genes in S. aureus	32
pUT18	Vector for two-hybrid analyses. Enables C-terminal fusion of T18 domain of adenylate cyclase to protein of interest	36
pUT18C	Vector for two-hybrid analyses. Enables N-terminal fusion of T18 domain of adenylate cyclase to protein of interest	36
pKT25	Vector for two-hybrid analyses. Enables N-terminal fusion of T25 domain of adenylate cyclase to protein of interest	36
pKNT25	Vector for two-hybrid analyses. Enables C-terminal fusion of T25 domain of adenylate cyclase to protein of interest	36

### Transcriptome analysis.

Triplicate cultures of SH1000 and a NIS-resistant derivative (SH1000 NsaS_A208E_) were grown at 37°C with aeration in MHB to an optical density of 0.2 at 600 nm. Two culture volumes of RNAprotect (Qiagen) were added to each culture, and the mixture was processed according to the manufacturer’s instructions. Processed cultures were incubated with lysostaphin (200 mg/liter) for 90 min at 37°C, followed by the addition of proteinase K (40 mg/liter) and incubation for a further 10 min at room temperature. Total RNA was purified using the RNeasy midikit (Qiagen).

Removal of rRNA from the samples, library creation, and RNAseq were performed at the Leeds Clinical Molecular Genetics Centre (St. James’ Hospital, University of Leeds) using the NextSeq platform (Illumina). Sequencing data were analyzed using CLC Genomics Workbench version 8 (Qiagen). Briefly, reads were trimmed and gene expression values for each sample replicate were calculated using the annotated sequence of S. aureus 8325 (accession number NC_007795) as a reference. Quality control for each sample was carried out using principal component analysis prior to quantile normalization (31). Relative expression values between groups (SH1000 versus SH1000 NsaS_A208E_) were subsequently calculated, and the significance of each value was determined by *t* test.

For RT-qPCR, superscript II reverse transcriptase (Invitrogen) was used to convert RNA to cDNA and levels of *vraD* and/or *braD* in each sample were determined by qPCR and ΔΔ*C_T_* analysis using the QuantiTect SYBR Green PCR kit (Qiagen) with appropriate oligonucleotide primers (see Table S1 in the supplemental material).

10.1128/mSphereDirect.00633-18.1 TABLE S1 Oligonucleotide primers used in this study. Download Table S1, DOCX file, 0.02 MB. Copyright © 2018 Randall et al. 2018 Randall et al. This content is distributed under the terms of the Creative Commons Attribution 4.0 International license.

### Gene inactivation and complementation studies.

Insertional inactivation of *nsaS* was achieved using the suicide vector pMUTIN4 (32), containing an ∼0.5-kb PCR-generated fragment comprising nucleotides 195 to 682 of *nsaS*.

For gene deletions, 1-kb regions of chromosomal DNA flanking the gene of interest were PCR amplified using Phusion DNA polymerase (NEB) and appropriate oligonucleotide primers (Table S1). PCR amplicons were introduced into the multiple cloning site of the allelic replacement vector pIMAY (33) by Gibson assembly, and the resulting constructs were used to transform E. coli SA08B (34) before recovery and electroporation into SH1000 NsaS_A208E_. Markerless deletion of the gene of interest was achieved as described previously (33). Complementation of gene deletions was achieved by expression of the gene of interest *in trans* from the anhydrotetracycline (ATc)-inducible expression vector, pRMC2 (35). Creation of a construct expressing BraD_E168Q_E was achieved using QuikChange site-directed mutagenesis (Agilent) on pRMC2:*braDE* using appropriate oligonucleotide primers (Table S1).

### Two-hybrid analysis of protein-protein interactions.

Two-hybrid analysis was carried out using the BACTH system (36). Genes encoding proteins of interest (POIs) were cloned into a suite of vectors to allow expression of POIs fused to the T25 (pUT18/pUT18C) or T18 (pKT25/pKNT25) domain of adenylate cyclase. T25 fusion constructs were cotransformed with T18 fusion constructs into E. coli BTH101 in all possible combinations and plated onto LBA containing IPTG and X-Gal. Transformants that turned blue following 48-h incubation at 30°C indicated a protein-protein interaction. A blue color observed for any combination of constructs for a given protein pair was considered evidence of protein interaction.

### Accession number(s).

Raw sequence reads and processed data are available from GEO and the SRA under accession number GSE114706.
