# Supplementary material for: Acquired Nisin Resistance in Staphylococcus aureus Involves Constitutive Activation of an Intrinsic Peptide Antibiotic Detoxification Module
Source: mSphere. 2018 Dec 12;3(6):e00633-18. doi: 10.1128/mSphereDirect.00633-18 (PMC6291627; doi:10.1128/mSphereDirect.00633-18)
Supplement: TABLE S1 [file sph006182723st1.docx]

| **Name** | **Sequence** | **Function** |
| --- | --- | --- |
| *nsaS* pMUTIN4-F | AGAACGAC**AAGCTT**ACAAATTGAAAACTTAGAAAATGC | Generation of construct insertional inactivation of *nsaS* |
| *nsaS* pMUTIN4-R | ATTGTATA**GGATCC**CTTCACTAATACCGATACCATTATC |  |
| *braDE* L pIMAY-F | ccctcgaggtcgacggtatcgataagcttgatatcgaattcctgcagcccTACTCAGGAAATAGCAATAG | Generation of markerless deletion constructs |
| *braDE* L pIMAY-R | tgattgtgatttcactgaaacatggTTCTTTGTTAGTTCTATATTAAA |  |
| *braDE* R pIMAY-F | gttttaatatagaactaacaaagaaCCATGTTTCAGTGAAATCAC |  |
| *braDE* R pIMAY-R | gagctccaccgcggtggcggccgctctagaactagtggatcccccACTATGGATTAAAGAGCTGC |  |
| *vraDE* L pIMAY-F | ccctcgaggtcgacggtatcgataagcttgatatcgaattcctgcagcccCTACAATGTCTGAAAATCATTTAACTTTAAC |  |
| *vraDE* L pIMAY-R | atgaagcatcttttaatcgtaagtgGTATTTAATTTCATTTTAAATAATGTTTGGA |  |
| *vraDE* R pIMAY-F | cattatttaaaatgaaattaaatacCACTTACGATTAAAAGATGC |  |
| *vraDE* R pIMAY-R | gagctccaccgcggtggcggccgctctagaactagtggatcccccCCATTTATTGTATTCTATAATCA |  |
| *vraD* pRMC2-F | ATCCATAC**GGTACC**AAAAGGAGTGAGACTATGACG | Generation of gene over-expression constructs |
| *vraD* pRMC2-R | CTAATAGC**GAGCTC**TGAAAACGATATGGTTAAATG |  |
| *vraE* pRMC2-F | ATCCATAC**GGTACC**CTTCAATCAGTATTAGGTGGTG |  |
| *vraE* pRMC2-R | CTAATAGC**GAGCTC**TGAAGCATCTTTTAATCGTAA |  |
| *vraDE* pRMC2-F | ATCCATAC**GGTACC**AAAAGGAGTGAGACTATGACG |  |
| *vraDE* pRMC2-R | CTAATAGC**GAGCTC**TGAAGCATCTTTTAATCGTAA |  |
| *braD* pRMC2-F | ATCCATAC**GGTACC**TAACAAAGAAATGAGGTGCA |  |
| *braD* pRMC2-R | CTAATAGC**GAGCTC**ATGTGCCATAACAATCTTAACT |  |
| *braE* pRMC2-F | ATCCATAC**GGTACC**CTTAGCACTTGTGAATGGAG |  |
| *braE* pRMC2-R | CTAATAGC**GAGCTC**GTGATTTCACTGAAACATGG |  |
| *braDE* pRMC2-F | ATCCATAC**GGTACC**TAACAAAGAAATGAGGTGCA |  |
| *braDE* pRMC2-R | CTAATAGC**GAGCTC**GTGATTTCACTGAAACATGG |  |
| BraD_E168Q_-F | GTTTTTGAATCAAGTGCACCTGTAGGTTGATCGGCTAG | Site directed mutagenesis of *braD* (generating BraD_E168Q_) |
| BraD_E168Q_-R | TCAACCTACAGGTGCACTTGATTCAAAAACATCAAAGGC |  |
| *braD* qRT-PCR-F | CGGCGGATACTTACTTGG | Quantitative measurement of gene expression by qRT-PCR |
| *braD* qRT-PCR-R | CTACAGAGTCAAATGGATAATACC |  |
| *vraD* qRT-PCR-F | GCACTAACTATGGCTATGACATC |  |
| *vraD* qRT-PCR-R | TGAATACAACATCGGTAATAGATACG |  |
| 16S qRT-PCR-F | ACGTGGATAACCTACCTATAAGACTGGGAT |  |
| 16S qRT-PCR-R | TACCTTACCAACTAGCTAATGCAGCG |  |
| *nsaS* 2H-F | TTAGCTAT**TCTAGA**GATGACCTTTCTTAAAAGTATTACTCAG | Generation of plasmids for two-hybrid analyses |
| *nsaS* 2H-R | TTCATAGC**GGTACC**CGTTCATCTGGAAATTGAATCGTA |  |
| *nsaR* 2H-F | TTAGCTAT**TCTAGA**GATGAAAATATTAATTGTTGAAGATGATTTT |  |
| *nsaR* 2H-R | TTCATAGT**GGTACC**CGTACTTTATATCCGACATTTTTCTTTGTAA |  |
| *vraD* 2H-F | TTGCTCCC**TCTAGA**GATGACGATATTATCAGTGCAACAT |  |
| *vraD* 2H-R | TTCATAGC**GGTACC**CGTTAAATGTCATTTGAGACACCAC |  |
| *vraE* 2H-F | TTGCTCCC**TCTAGA**GATGACATTTAACCATATCGTTTTC |  |
| *vraE* 2H-R | TTCATAGC**GGTACC**CGTTAAATGGTTTTCTTAATCAATTTG |  |
| *braD* 2H-F | TTGCTCCC**TCTAGA**GATGACGATATTATCAGTGCAACAT |  |
| *braD* 2H-R | TCTTAGCT**GGTACC**CGAATGTCATTTGAGACACCACC |  |
| *braE* 2H-F | TTAGCTAT**TCTAGA**GATGACATTTAACCATATCGTTTTC |  |
| *braE* 2H-R | TTCATAGC**GGTACC**CGAATGGTTTTCTTAATCAATTTGTTT |  |

Nucleotides shown in bold type face represent engineered restriction sites

Nucleotides in lower case represent regions of homology for Gibson assembly
